# Trained to care, untrained to share: the integration of social media (#SoMe) education in dental specialty programs: a scoping review

**DOI:** 10.3389/froh.2025.1700491

**Published:** 2026-01-12

**Authors:** Noha Taymour, Ayman Raouf Khalifa, Hams H. Abdelrahaman, Mohamed G. Hassan

**Affiliations:** 1 Department of Substitutive Dental Sciences, College of Dentistry, Imam Abdulrahman Bin Faisal University, Dammam, Saudi Arabia; 2 Department of Preventive Dentistry, Gulf Medical University, Ajman, United Arab Emirates; 3 Department of Pediatric Dentistry, and Dental Public Health, Faculty of Dentistry, Alexandria University, Alexandria, Egypt; 4 Division of Bone and Mineral Diseases, Department of Internal Medicine, School of Medicine, Washington University in St. Louis, St. Louis, MO, United States; 5 Department of Orthodontics, Faculty of Dentistry, Assiut University, Assiut, Egypt

**Keywords:** social media, dental education, e-professionalism, digital literacy, ethics, curriculum development

## Abstract

**Background:**

Social media (SoMe) is increasingly used in dentistry for patient education, professional networking, and career development, yet formal curricula on its use and digital literacy remain limited in dental specialty programs.

**Aim:**

This scoping review the existing literature on SoMe use in dentistry and identify gaps in curricular implementation and policy development for curricular integration in dental specialty training.

**Methods:**

Following PRISMA- ScR guidelines, a systematic literature search of PubMed and Scopus for studies published between 2009 and 2024 were searched. Two reviewers identified 2,952 articles, of which 531 met inclusion criteria. Data were extracted and analyzed to identify publication trends, thematic areas, and key findings related to SoMe in dentistry.

**Results:**

Research output grew substantially (412%) between 2015 and 2024. YouTube was the most studied platform (41%), followed by X/Twitter (27%), Instagram (19%), and Facebook (13%). Research involving dental professionals (54%) emphasized continuing education and networking, while patient-focused studies (43%) addressed oral health promotion, misinformation, and treatment decision-making. Only 7% evaluated formal SoMe curricula. Reported challenges, privacy breaches (38%), unprofessional conduct (32%), and misinformation (29%), highlights the need for structured educational content during dental specialty training.

**Conclusion:**

SoMe changed the dynamics of clinical dental practice; however, concerns persist regarding digital literacy and professionalism. Evidence on the structured integration of SoMe into dental specialty curricula remains limited, highlighting the need for educational initiatives to promote ethical digital engagement and oral health communication.

## Graphical Abstract

**Figure:**
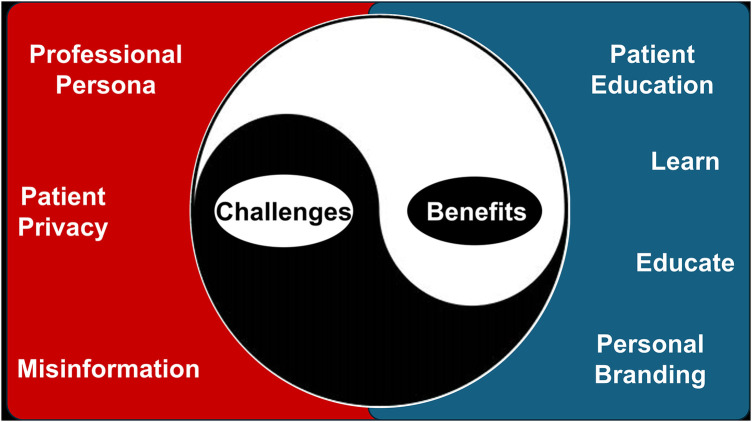
Yin-yang symbol with Challenges in the black section and Benefits in the white section. The left red background highlights Professional Persona, Patient Privacy, and Misinformation. The right blue background emphasizes Patient Education, Learn, Educate, and Personal Branding.

## 1 Introduction

Social media (SoMe) has become an integral component of healthcare communication, transforming how professionals and patients access, share, and discuss health information (1). Platforms such as Instagram, Facebook, X (formerly Twitter), YouTube, and TikTok have emerged as influential tools for professional development, patient education, and public health advocacy (2–4). In dentistry, these platforms have revolutionized clinical outreach, oral health promotion, and peer-to-peer learning, broadening opportunities for both professional collaboration and patient engagement (5).

However, this digital transformation has brought new challenges, including the spread of misinformation, breaches of patient confidentiality, and uncertainty surrounding professional boundaries (5–8). These risks highlight the urgent need for clear ethical frameworks and formal training on the responsible use of SoMe. Despite its growing role in healthcare education, structured curricula addressing digital professionalism and evidence-based online communication remain scarce in dental specialty programs. Consequently, residents are often underprepared to navigate the opportunities and risks of digital engagement, potentially undermining patient trust and professional credibility.

The responsible and strategic integration of SoMe into dental education is increasingly viewed as essential for cultivating digital professionalism as we stepping into a new era of clinical dentistry (9, 10). This includes the capacity to communicate, educate, and advocate effectively while maintaining ethical and professional standards. Developing this competency requires educational institutions to establish formal frameworks for SoMe use, balancing innovation with professionalism and patient protection.

Despite the growing attention to SoMe in dental education, there is still limited evidence on how formal SoMe training need to be integrated into specialty curricula and how it could influence professionalism, communication, and patient engagement. Therefore, this scoping review aims to address the following research question: “What is the current state of evidence regarding the use, benefits, challenges, and educational integration of social media in dental specialty programs?” Specifically, the review seeks to: 1) Map existing literature on SoMe use in dentistry; 2) identify gaps in curricular implementation and policy development; and 3) propose actionable recommendations for integrating structured SoMe education into dental specialty training. By synthesizing available evidence and highlighting unmet educational needs, our findings contribute to the evolving discourse on digital professionalism in dentistry. This review provides guidance for educators, professional organizations, and policymakers to enhance SoMe literacy, promote ethical online engagement, and strengthen the dental profession's credibility in an increasingly digital healthcare landscape.

## 2 Methods

This scoping review was conducted in accordance with the Arksey and O'Malley framework, to systematically map the existing literature on the integration of social media (SoMe) in dental specialty programs. The review aimed to identify key concepts, research gaps, and types of evidence available in this field.

### 2.1 Search strategy

To enhance conceptual clarity, we applied the PCC (Population–Concept–Context) framework recommended for scoping reviews under the PRISMA-ScR guidelines. The Population (P) included dental residents, faculty, and institutions involved in postgraduate or specialty dental education. The Concept (C) focused on the use and integration of social media (SoMe) in educational or professional training. The Context (C) focused on dental specialty programs across clinical disciplines and educational settings worldwide. This framework guided eligibility criteria, search strategy, and data extraction without restricting study design or outcomes. Search was conducted in PubMed, Web of Science and Scopus (coverage: 2009 to March 2025) using the following query syntax: Pubmed: ((“social media"[Title/Abstract] OR “Facebook"[Title/Abstract] OR “Instagram"[Title/Abstract] OR “Twitter"[Title/Abstract] OR “YouTube"[Title/Abstract] OR “TikTok"[Title/Abstract] OR “LinkedIn"[Title/Abstract] OR “Snapchat"[Title/Abstract] OR “social networking sites"[Title/Abstract] OR “online platforms"[Title/Abstract]) AND (“dentistry"[Title/Abstract] OR “dental"[Title/Abstract] OR “orthodontics"[Title/Abstract] OR “endodontics"[Title/Abstract] OR “periodontics"[Title/Abstract] OR “prosthodontics"[Title/Abstract] OR “oral surgery"[Title/Abstract] OR “dental education"[Title/Abstract] OR “dental practice"[Title/Abstract])). Search strings were adapted for database-specific indexing. All records were exported on March 1, 2025 to an Excel Spreadsheet, and imported into reference manager software for deduplication.

### 2.2 Eligibility criteria

Inclusion Criteria including review articles, original research, and qualitative studies focusing on the use of SoMe in dentistry, articles published in English. Exclusion were grey literature, editorials, commentaries, conference abstracts, and opinion pieces without empirical data. Studies are not directly related to dentistry or dental education or not available in English.

### 2.3 Study selection

Two reviewers (H.H.A., M.G.H.) independently screened titles and abstracts (*n* = 2,952) and subsequently full texts (*n* = 531) against predefined inclusion/exclusion criteria. Disagreements were resolved by consensus with a third reviewer (N.T.). Data-extraction fields included study design, specialty, SoMe platform, target population, and educational outcomes. A PRISMA flow diagram (Graphical Abstract) illustrates the study selection process, detailing the number of records identified, screened, and included.

### 2.4 Data extraction and reporting

Data extraction was performed using a standardized form, capturing information on publication year, study design, dental specialty, SoMe platform, target population and main findings related to benefits and challenges, and ethical considerations or educational recommendations. Descriptive statistics summarized study characteristics; thematic analysis identified recurrent benefits, challenges, and educational gaps to identify overarching trends and patterns.

## 3 Results

**Figure 1:**
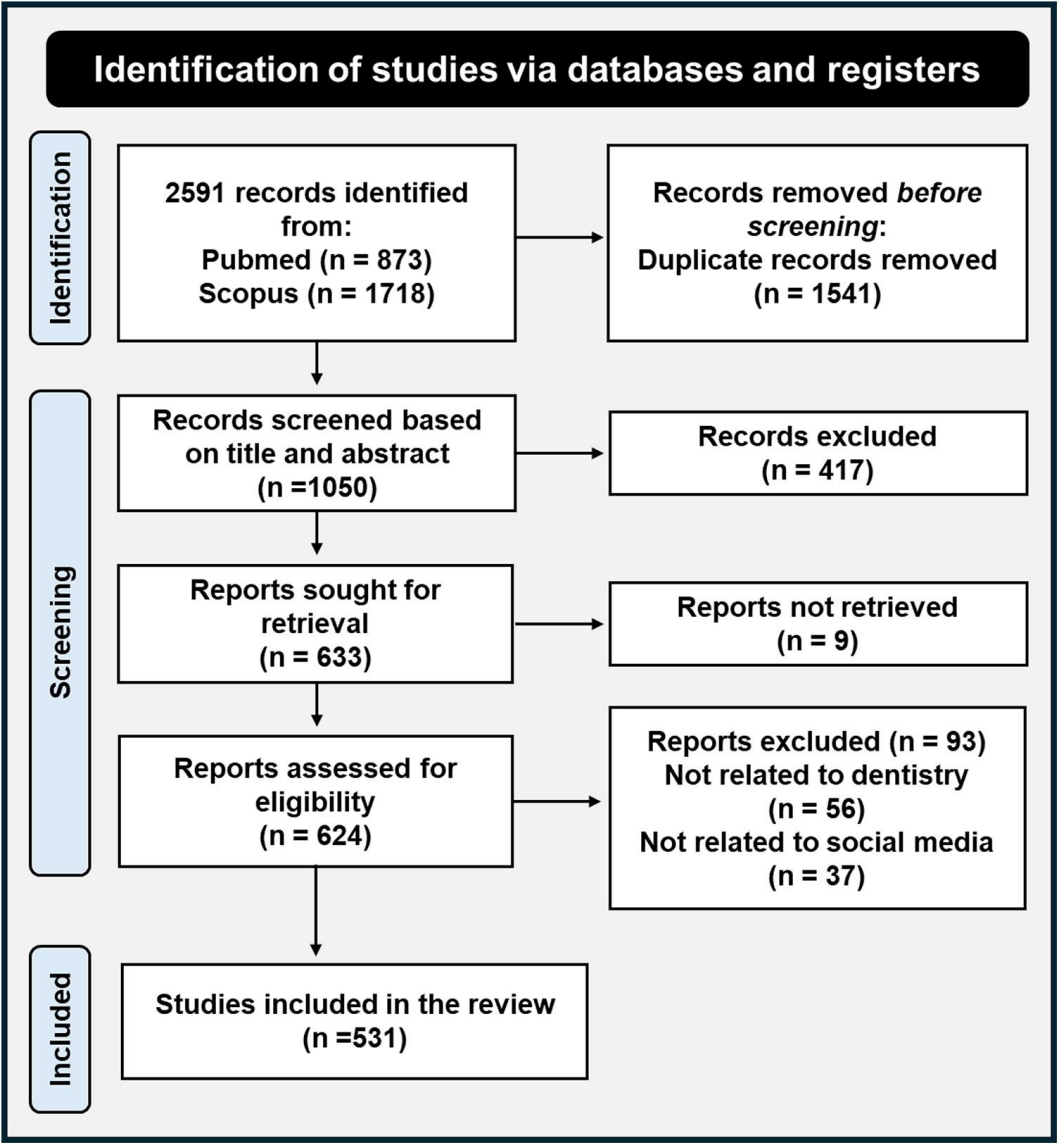
PRISMA flow diagram.

Following the systematic search and screening process, a total of 531 articles were included in this scoping review. The detailed selection process is outlined in Figure 1 (PRISMA Flow Diagram). The included studies comprised a variety of research designs, with a predominance of descriptive studies and reviews.

### 3.1 Emerging roles of SoMe in dental practice: dentistry is online

#### 3.1.1 Publication trends and scholarly interest

**Figure 2:**
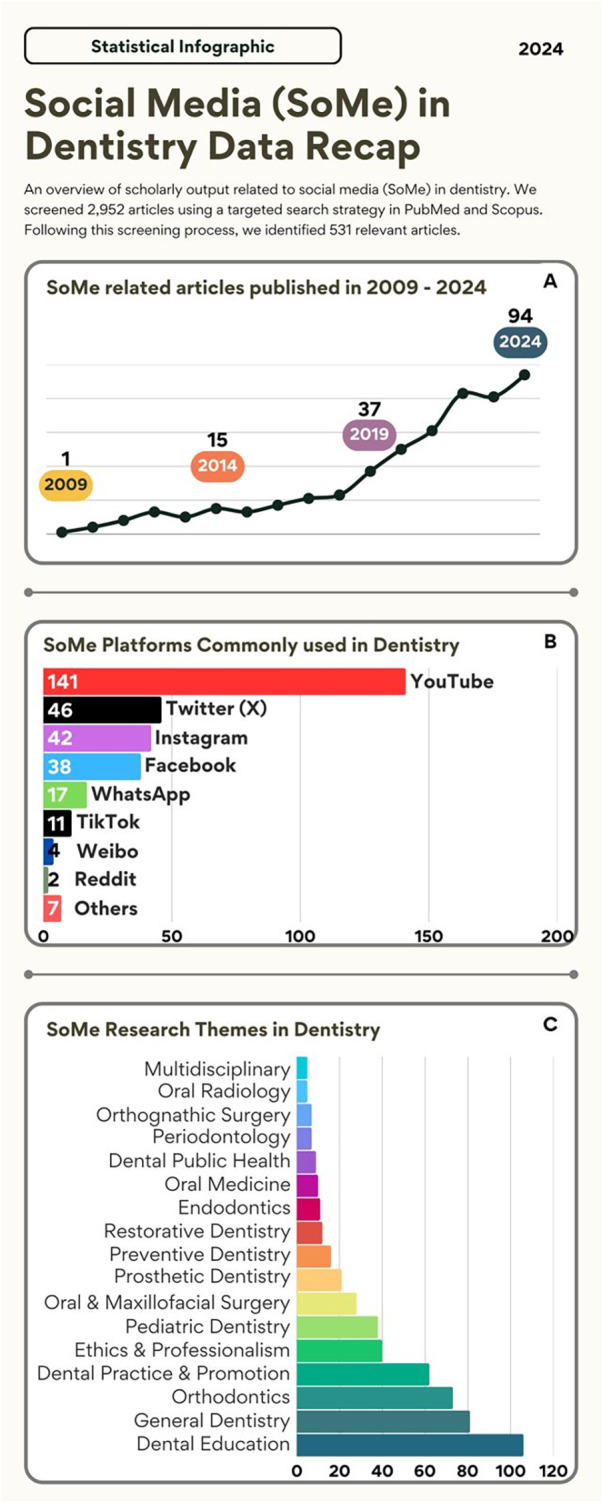
Social media (SoMe) in dentistry data recap. **(A)** SoMe related articles in 2009-2024. **(B)** SoMe platforms commonly used in dentistry. **(C)** SoMe research themes in dentistry.

The rapid spread of SoMe in dentistry highlights its role as an effective channel for patient education, professional networking, and professional continuous education. Platforms, like YouTube, TikTok and Instagram, are not only transforming traditional patient care models but also reshaping how dentists engage with their communities and advance their clinical practices (11). The data in Figure 2A illustrates the temporal distribution of published articles from 2009 to 2024, highlighting a significant increase in research output over time. In the initial years (2009–2013), the number of published articles remained relatively low, with minimal annual variations. Between 2009 and 2013, the number of articles ranged from 1 to 13 per year, collectively accounting for a small proportion of the total research output. A moderate increase was observed between 2014 and 2018, during which the number of published articles exhibited a steady upward trend, rising from 15 in 2014 to 23 in 2018. This reflects an increase in scholarly interest in SoMe in the dental field, which may be due to a greater recognition of its relevance. The number of articles increased substantially from 37 in 2019 to 94 in 2024, representing a more than two-fold increase within five years. This is particularly evident between 2020 and 2022 when publications rose from 50 in 2020 to 83 in 2022. The peak in 2024, with 94 articles accounting for 17.7% of the total, suggests a sustained expansion of interest in the topic (Figure 2A).

#### 3.1.2 Target populations in SoMe research

Among 531 articles, dentists and dental students were the most targeted population, featured in 285 articles (Supplementary Figure S1). Research in this category often explores professional communication, continuing education, clinical practice and promotion, and the ethical implications of social media use in dentistry. Patients were the focus of 226 studies, with research themes centering on oral health awareness, oral health misinformation, and the role of SoME in influencing treatment decisions and choosing dental practice (Supplementary Figure S1). A smaller subset of 20 articles targeted both dentists and patients covering patient-dentist communication via social media platforms and shared perspectives on digital oral health content (Figure 2B).

#### 3.1.3 Impact of COVID-19 on SoMe use in dentistry

Five years ago, the COVID-19 pandemic spotted the light on the role of SoMe in dental professional and patient education (12, 13). With the disruption of dental services, SoMe platforms emerged as vital tools for delivering oral health information and sustaining patient engagement.

### 3.2 Thematic areas of SoMe research in dentistry

The analysis of social media (SoME) research in dentistry reveals a strong emphasis on its role in dental education, with 106 studies exploring its applications in teaching, learning, and professional development. This reflects the increasing integration of social media as a tool for knowledge dissemination, virtual learning, and student engagement. Following dental education, general dentistry (81 studies) and orthodontics (73 studies) emerged as major areas of focus (Figure 2C).

Research in these fields often examines patient education, professional discussions, and the dissemination of evidence-based information through social media platforms. Additionally, dental practice and promotion were a key theme in 62 studies, highlighting the role of social media in patient outreach, clinic marketing, and reputation management. Studies in pediatric dentistry (38 studies) further underscore the potential of social media in educating parents and caregivers about children's oral health (Figure 2C).

## 4 Discussion

The findings of this scoping review highlight the growing and evolving role of SoMe in dentistry, extending beyond mere communication to encompass patient education, professional development, and public health promotion. SoMe dental-related research output has expanded substantially over the past decade, reflecting an accelerating recognition of its relevance to clinical education and practice. This trend aligns with broader transformations in healthcare communication, where digital literacy and professionalism have become essential competencies for future practitioners (9, 10).

**From platforms to concepts:**

SoMe platforms were employed either as an intervention tool or for content analysis. YouTube was the most frequently used platform, appearing in 141 studies followed by Twitter (X) in 46 studies, Instagram in 42 studies, and Facebook with 38. WhatsApp and TikTok were the least represented with 17 and 11 studies, respectively. This distribution could reflect different academic interests and the applicability of such platforms in the dental field (Figure 2B). These platforms are valuable, cost-effective tools for enhancing professional and/or therapeutic oral health knowledge. Their potential in public health initiatives would be beneficial in campaigns targeting oral hygiene education, which leverage the widespread reach and accessibility of these platforms to engage laypersons effectively (14). YouTube is used extensively in dental education, providing extensive video-based resources on oral health, clinical procedures, and professional development. Patients benefit from instructional videos that enhance their understanding of dental treatments and preventive care measures (15–17), while dental students and dental professionals use YouTube to access recorded material to learn surgical and clinical skills (18–23). SoMe platforms, like Instagram, have proven to be valuable venues used for visual communication in dentistry, offering a medium for disseminating oral health information and clinical insights. Patients engage with content related to preventive care, dental treatment, and aesthetics (24–27). It boosts patient enthusiasm and satisfaction in long-term treatments like orthodontic interventions (28).

Besides patient education and communication, SoMe has been extensively used in marketing dental products, services, and personal branding (29–32). A well-planned SoMe strategy enables dental practices to maintain a consistent online presence by sharing content tailored to engage their target audience. Platforms such as Instagram and TikTok are particularly effective for showcasing clinical expertise, patient testimonials, and educational materials, thereby fostering trust and expanding outreach (33, 34). SoMe analytics further enhance these efforts by providing detailed insights into patient demographics and preferences, enabling more precise and impactful marketing strategies (35). Moreover, these platforms allow dental specialists to engage directly with patients by responding promptly to inquiries, addressing concerns, and offering guidance, which can influence patient decisions when selecting a provider (36–38). Finally, SoMe has significantly transformed professional networking in dentistry. Platforms such as LinkedIn and specialized dental forums provide valuable opportunities for dentists to connect with colleagues, exchange knowledge, and remain informed about industry advancements (39). Twitter (X) and Facebook also could help as a dynamic platform for real-time knowledge exchange, public health advocacy, and professional discourse. These platforms can provide access to academic study groups and institutional updates (40–43). These online communities facilitate the sharing of complex cases, seeking advice from specialists, and discussing emerging techniques and technologies, fostering collaboration and enhancing patient care (5). To sum up, SoMe has become a reflection of the profession's digital maturity. Rather than focusing solely on where dentists engage, attention must shift to how they engage—ethically, educationally, and transparently. Embedding SoMe literacy within dental specialty programs can strengthen digital professionalism, counter misinformation, and enhance patient-centered communication. This conceptual approach reframes SoMe not as a collection of tools but as a critical educational domain that defines the future of professional integrity in dentistry.

### 4.1 Social media literacy matters for today's dental professionals

The use of SoMe in dentistry embodies the Yin Yang principle, where seemingly opposing forces are interconnected and interdependent. On one side, SoMe offers numerous benefits for dental practices, such as oral health promotion, patient education, and professional networking. These aspects represent the Yin side, focusing on relationship building, sharing, and engaging with the community. This approach fosters a sense of connection and trust between dental professionals and their patients, enhancing practice quality and patient care. On the opposite side, it also presents significant challenges and potential risks, such as ethical dilemmas, professional misconduct, and patient confidentiality breaches, these challenges embody the Yang side, emphasizing the need for technological vigilance, analytical thinking, and adherence to strict guidelines (Graphical Abstract).

A recently published scoping review highlights many of these challenges like the prevalence of health misinformation, noting that users often lack the necessary health and digital literacies to discern credible information (44). One prominent challenge is the violation of patient privacy (5). Despite its vast potential for outreach and education, concerns about patient privacy, obtaining consent, and the misuse of clinical images or patient data are raised. SoMe inherently carries the risk of accidental disclosure of protected health information (PHI), which can lead to breaches of privacy and violations of laws like HIPAA (Health Insurance Portability and Accountability Act) (45). A recent analysis of dental-related social media content showed that clinical cases comprise the largest share of content, followed by patient education materials, professional development resources, and practice promotion (46). Moreover, a recent study found that 38% of dental professionals had inadvertently posted content that could compromise patient privacy, highlighting a gap in understanding and adherence to legal and ethical guidelines (47). These breaches not only jeopardize patient trust but can also result in legal consequences, professional sanctions, and reputational damage (48). This pattern has been reported across different medical disciplines, where 46.6% of posts were likely identifiable by patients, and 2% included patients' names (49). For dental professionals, ensuring HIPAA compliance when using SoMe is critical but challenging, given the public and informal nature of these platforms. Formal SoMe education should be mandatory to use social media tools effectively while protecting patient trust by following legal and ethical standards.

Another significant issue is the spread of misinformation. While SoMe enables dental professionals to share expertise, it could serve as a platform for nonfactual or pseudoscientific claims. Unverified claims about the efficacy and safety of different dental treatments can mislead patients into pursuing inappropriate options without professional consultation (7, 8, 27, 50). For example, a recent survey revealed that 91% of orthodontic residents consider social media effective for orthodontic advertising, yet 72% admitted they do not share evidence-based content on these platforms (51). This controversy brings attention to an important issue that must be addressed. The rise of direct-to-consumer clear aligner services, heavily advertised online, has raised serious concerns within the field. Previously, we had shown that, although dental professionals produce most of the clear aligner content on Instagram, much of it is self-promotional and lacks accurate information (27). The same low quality of clear aligner content was seen across different social media platforms (52, 53). Similarly, the promotion of dental implants over social media without emphasis on necessary clinical evaluations can lead to complications. The analysis of implant-related content on Instagram showed that although 42% of the content was created by dental professionals, only 27% of the content was supported with factual claims (8). Dental specialists must be aware of sharing evidence-based information and remind the patients about the personalized nature of dental treatments to counteract the spread of misinformation and safeguard our profession.

The maintenance of a professional online persona is one of the complex challenges dental professionals faces while using digital platforms. SoMe can blur the lines between personal and professional boundaries, leading to unprofessional conduct by dental practitioners (54). Dental professionals' SoMe activities often caught the attention of different groups of interest, including personal posts, which can affect their professional reputation. The General Dental Council's guidance emphasizes the importance of separating personal and professional identities online to avoid reputational harm (55). A recent survey among students in orthodontic residency programs revealed that although the majority of the residents use social media platforms for several professional reasons, only 37% of the participants had separate accounts for personal and professional use (51). The digital fingerprint of dental professionals might affect professional reputation (55, 56). Recent study highlighted the role of physician-rating websites as a potential source of information about the quality of health care from the patient's perspective (57). Therefore, experts are calling for better education, ethical guidelines, and tools to help dental professionals navigate social media responsibly while maintaining their credibility (58–61). Formal social media education can provide dental residents with strategic and ethical approaches to maintaining a digital fingerprint that not only enhances a dentist's professional image but also contributes to better patient engagement and education.

### 4.2 Benefits of SoMe education in dental specialty training programs

As dentistry increasingly recognizes social media's potential, structured training can ensure its responsible use to combat misinformation, amplify research dissemination, and foster lifelong learning. There is increasing evidence supporting the positive impact of structured training programs in improving awareness and online behavior, emphasizing their role in shaping responsible digital professionalism (62, 63). The following discussion highlights four key benefits of incorporating social media education into medical residency programs, supported by evidence and research.

#### 4.2.1 Improved professionalism and ethical use

Social media education helps dental residents recognize ethical guidelines, ensuring adherence to professional conduct and HIPAA regulations. Awareness of social media risks among dental students is generally high. Studies reveal an understanding of potential reputational damage, privacy breaches, and legal ramifications (64, 65). Yet, gaps in knowledge persist, particularly concerning the nuances of legal and ethical guidelines, such as HIPAA regulations in the United States or the General Data Protection Regulation (GDPR) in Europe (66). Comprehensive training programs can address these gaps, equipping students with the tools to navigate complex online interactions responsibly. Residents can be trained to avoid sharing patient data, posting inappropriate content, or engaging in unprofessional behavior online. This education will be beneficial when they establish their own practices, helping them avoid malpractice issues and ensure compliance with HIPAA guidelines.

#### 4.2.2 Enhanced communication skills

SoMe platforms as powerful tools for patient engagement, offer unlimited opportunities to foster transparency, trust, and education. By mastering these digital communication channels, residents can create targeted educational content that addresses patient concerns, simplifying complex dental procedures, and promotes oral health literacy. The ability to identify and counteract health-related misinformation becomes crucial, as residents learn to distinguish between evidence-based information and potentially harmful online narratives. Training programs that incorporate social media communication skills enable residents to develop strategic content-creation techniques, transforming complex medical information into accessible, patient-friendly formats. Moreover, these platforms facilitate global professional networking, allowing residents to connect with peers, mentors, and experts, thereby expanding their knowledge and collaborative potential beyond traditional academic boundaries. The shift towards digital communication requires residents to understand the nuanced ethical considerations of online patient interactions, including maintaining patient confidentiality and professional boundaries. Effective social media training equips dental professionals with the skills to create engaging, informative content that not only educates patients but also builds their practice's digital reputation.

#### 4.2.3 Building a professional brand

Building a professional brand has become important for dental residents in the digital era, with SoMe platforms offering unique opportunities for career development and professional growth. By strategically showcasing their achievements, sharing comprehensive clinical cases, and actively engaging with academic communities online, residents can significantly enhance their visibility within their field. This digital presence not only highlights their expertise but also demonstrates their commitment to ongoing learning and professional development. SoMe platforms enable residents to connect with established leaders in dentistry, creating valuable mentorship relationships and opening doors to learning opportunities that might otherwise be inaccessible. These connections can provide new aspects of cutting-edge techniques, research opportunities, and career advice, accelerating professional growth. Moreover, by consistently sharing high-quality, evidence-based content and engaging in meaningful discussions, residents can gradually establish themselves as reliable sources of dental knowledge. This process of building thought leadership not only benefits their current standing but also lays the groundwork for future career advancement. As they develop a strong online presence, residents can position themselves as emerging leaders in their specialties, potentially attracting collaborations, speaking engagements, and career opportunities. However, it's crucial for residents to maintain a balance between professional branding and ethical considerations, ensuring that their online activities align with professional standards and patient confidentiality guidelines.

#### 4.2.4 Enhancing patient-centered care

Enhancing patient-centered care through social media enables dental residents to gain valuable insights into patient perspectives, ultimately improving the quality of care they provide. By actively engaging with patients on social media platforms, residents can stay attuned to their concerns, needs, and experiences in real time (67). This direct line of communication allows for a more nuanced understanding of patient expectations and preferences, which is crucial for delivering personalized care. Social media also provides a platform for patients to share their healthcare journeys, offering residents a unique window into the patient experience beyond clinical encounters (68). This enhanced understanding can lead to more empathetic and tailored care approaches. Furthermore, social media facilitates the creation of online communities where patients can find support and share experiences, empowering them to take a more active role in their healthcare (69). By monitoring these interactions, residents can identify common issues and address them proactively in their practice. The integration of social media into patient-centered care strategies not only improves communication but also provides a sense of trust and transparency between healthcare providers and patients (70). This approach aligns with the growing trend towards consumer-driven healthcare, where patients are increasingly involved in their care decisions and expect more personalized attention from their providers.

Consequently, the incorporation of SoMe literacy into dental residency curricula could limit several risks by equipping residents with the knowledge to navigate digital platforms responsibly. By implementing a culture of digital professionalism, residency programs can transform SoMe from a liability into a powerful tool for patient education and professional collaboration, ensuring both ethical integrity and compliance with privacy standards.

This scoping review, while comprehensive, is subject to certain limitations, the inherent heterogeneity of study designs and reporting across the included articles limited the ability to conduct a meta-analysis or draw definitive causal conclusions. Additionally, the rapid evolution of social media platforms means that findings may quickly become outdated, necessitating continuous updates to the literature.

## 5 Actions plan for integrating SoMe education into dental specialty training

In the previous sections, we covered how social media is important in dental practice. In addition, we discussed the common challenges faced by practitioners, including maintaining patient confidentiality, combating misinformation, and adhering to professional guidelines. The following recommendations outline practical strategies for integrating SoMe education into dental specialty training, ensuring residents maximize its benefits while upholding the highest professional and ethical standards.

### 5.1 Dental educational institutions

Specialty programs should start creating a structured SoMe curriculum as part of obligatory courses required for graduation. It is essential to ensure that dental residents understand both the opportunities and responsibilities of using digital platforms as part of developing their critical thinking (71). The curriculum should include modules that cover platform-specific functionalities, digital communication strategies, professional conduct, ethical use, HIPAA compliance, patient privacy, and the role of social media in patient education and professional networking. Programs can utilize a blended learning approach, combining online resources with in-person workshops to maximize engagement and learning outcomes. Moreover, residency programs should implement interactive workshops focusing on case studies and real-world scenarios that can provide practical insights into effective social media use. Specialty-specific guidance on sharing case studies or engaging with evidence-based content could further enhance the responsible use of social media in fields like prosthodontics, orthodontics, and periodontics (48). By implementing a culture of digital professionalism, residency programs can transform social media from a liability into a powerful tool for patient education and professional collaboration, ensuring both ethical integrity and compliance with privacy standards. Such scenario-based activities encourage actionable steps toward safeguarding personal and professional boundaries, showcasing the immediate practical outcomes of such training (66, 72). In addition, assessment tools such as self-assessment questionnaires and performance tasks can evaluate residents' understanding and application of social media skills. For instance, some medical institutions like Johns Hopkins have implemented social media professionalism workshops for medical trainees (73).

### 5.2 Professional organizations

Dental professional societies should establish and regularly update best practice guidelines for social media use, similar to the approach taken by the Hong Kong Academy of Medicine (HKAM) (74). Moreover, the incorporation of continuing education courses and workshops on effective social media strategies as part of specialty license renewal can equip dental practitioners with essential skills in digital content creation, patient engagement, and reputation management. Interventional workshops, such as those leveraging the Medical Education e-Professionalism (MEeP) framework, have proven effective in improving attitudes, subjective norms, and perceived behavioral control related to professional conduct on social media (75, 76). These workshops enhance not only the theoretical understanding of professionalism but also the intention to implement these principles in daily practice. Despite these advancements, studies indicate that many students continue to engage in potentially unprofessional behavior online, underscoring the need for continuous reinforcement of e-professionalism (77, 78). These initiatives not only promote professionalism but also help safeguard public trust by ensuring that online interactions align with ethical and regulatory standards.

### 5.3 Individuals

Dental specialists should prioritize ongoing self-assessment and personal auditing of their social media presence such as using YouTube for educational videos or Instagram for showcasing before-and-after cases. Additionally, individual practitioners should establish personal benchmark goals for professional social media engagement based on their specific practice needs and specialty requirements.

## 6 Conclusion

SoMe has become integral to modern dentistry, offering opportunities for education, communication, and professional networking. Yet, its responsible use requires structured training. Integrating SoMe education into dental specialty programs can strengthen digital professionalism, enhance patient engagement, and support ethical practice in an increasingly connected healthcare environment. This integration requires a coordinated, multi-level approach involving dental institutions, professional organizations, and specialists to foster digital professionalism and responsible online engagement.

## Data Availability

The original contributions presented in the study are included in the article/Supplementary Material, further inquiries can be directed to the corresponding authors.
