# Supplementary material for: Trained to care, untrained to share: the integration of social media (#SoMe) education in dental specialty programs: a scoping review
Source: Front Oral Health. 2026 Jan 12;6:1700491. doi: 10.3389/froh.2025.1700491 (PMC12832904; doi:10.3389/froh.2025.1700491)
Supplement: Supplementary file 1 [file Table1.docx]

**Supplemental table 1: Search Syntax**

| **Platform** | **Search strategy** |
| --- | --- |
| **PubMed** | **(("social media"[Title/Abstract] OR "Facebook"[Title/Abstract] OR "Instagram"[Title/Abstract] OR "Twitter"[Title/Abstract] OR "YouTube"[Title/Abstract] OR "TikTok"[Title/Abstract] OR "LinkedIn"[Title/Abstract] OR "Snapchat"[Title/Abstract] OR "social networking sites"[Title/Abstract] OR "online platforms"[Title/Abstract]) AND ("dentistry"[Title/Abstract] OR "dental"[Title/Abstract] OR "orthodontics"[Title/Abstract] OR "endodontics"[Title/Abstract] OR "periodontics"[Title/Abstract] OR "prosthodontics"[Title/Abstract] OR "oral surgery"[Title/Abstract] OR "dental education"[Title/Abstract] OR "dental practice"[Title/Abstract]))** |
| **Web of Science** | **TS=("social media" OR "Facebook" OR "Instagram" OR "Twitter" OR "YouTube" OR "TikTok" OR "LinkedIn" OR "Snapchat" OR "social networking sites" OR "online platforms") AND TS=("dentistry" OR "dental" OR "orthodontics" OR "endodontics" OR "periodontics" OR "prosthodontics" OR "oral surgery" OR "dental education" OR "dental practice")** |
| **Scopus** | **(TITLE-ABS-KEY("social media" OR "Facebook" OR "Instagram" OR "Twitter" OR "YouTube" OR "TikTok" OR "LinkedIn" OR "Snapchat" OR "social networking sites" OR "online platforms")) AND (TITLE-ABS-KEY("dentistry" OR "dental" OR "orthodontics" OR "endodontics" OR "periodontics" OR "prosthodontics" OR "oral surgery" OR "dental education" OR "dental practice"))** |
|  | **From 2009 to 2025** |
