# Supplementary material for: Trained to care, untrained to share: the integration of social media (#SoMe) education in dental specialty programs: a scoping review
Source: Front Oral Health. 2026 Jan 12;6:1700491. doi: 10.3389/froh.2025.1700491 (PMC12832904; doi:10.3389/froh.2025.1700491)
Supplement: Supplementary file 2 [file Table2.docx]

**Supplemental table 2: List of all included studies**

| **ID** | **Title** |
| --- | --- |
| **1** | Dangers and Benefits of Social Media on E-Professionalism of Health Care Professionals: Scoping Review |
| **2** | Instagram Dentistry |
| **3** | NHS dentistry: The social media challenge |
| **4** | The #ethics behind social media influencers in dentistry |
| **5** | Dentistry on TikTok - oh no |
| **6** | Digital and social media risks: perspectives on dental education and the profession |
| **7** | Social media in dental education: The need for institutional policies and content regulation |
| **8** | Orthodontics social media calling for help: How big is the problem? |
| **9** | International Journal of Paediatric Dentistry on Social Media |
| **10** | What are the ethical considerations of using video social media platforms such as TikTok in your dental practice? |
| **11** | Including social media and the ethical and legal debate in dental education |
| **12** | Scoping review on the role of social media in oral health promotion |
| **13** | What Social Media Platforms Should Be Used By A Dental Practice? |
| **14** | The Dental Press Journal of Orthodontics in the social media: a new interactive channel |
| **15** | Use of WhatsApp in Dental Education: a Scoping Review |
| **16** | Preparing dental students to use social media as a platform to promote oral health |
| **17** | Social media for the dental practice |
| **18** | Social media and orthodontics: Are our patients scrolling? |
| **19** | The role of Twitter in dental education: A systematic review |
| **20** | Facebook endodontic groups as potential tools to provide learning opportunities |
| **21** | Social media conversations about community water fluoridation: formative research to guide health communication |
| **22** | Qualitative Evaluation of YouTube Videos on Dental Fear, Anxiety and Phobia |
| **23** | Darwinian Dentistry? Social Media, Smartphones and Selfie Sticks |
| **24** | Social media and orthodontic treatment from the patient's perspective: a systematic review |
| **25** | Dental education and undue exposure of patients' image in social media: A literature review |
| **26** | Methodological Clarifications and Generalizing From Weibo Data. Comment on "Nature and Diffusion of COVID-19-related Oral Health Information on Chinese Social Media: Analysis of Tweets on Weibo" |
| **27** | Clicking on professionalism? Thoughts on teaching students about social media and its impact on dental professionalism |
| **28** | Social media and dentistry: can the new 'word of mouth' help your practice grow? |
| **29** | How to Get the Word Out that Your Dental Practice is on Social Media |
| **30** | The internet: education, social media and dental practice |
| **31** | The pattern and use of Twitter among dental schools in Saudi Arabia |
| **32** | Fake news and dental education |
| **33** | Public and dental professionals' use of social media to discuss amelogenesis imperfecta |
| **34** | Orthodontists on Social Media: Instagram's Influence |
| **35** | Antibiotics and toothache: a social media review |
| **36** | Esthetic Dentistry on Twitter: Benefits and Dangers |
| **37** | Social Media Usage among Dental Undergraduate Students-A Comparative Study |
| **38** | Orthodontic clear aligners and TikTok videos: A content, reliability and quality analysis |
| **39** | Social media as a learning tool: Dental students' perspectives |
| **40** | Nocturnal mouth-taping and social media: A scoping review of the evidence |
| **41** | Parallel endodontic education via social media: An exploratory survey study |
| **42** | The role of social media in dental education |
| **43** | Effect of Social Media on Patient's Perception of Dental Aesthetics in Saudi Arabia |
| **44** | YouTube Videos on Nutrition and Dental Caries: Content Analysis |
| **45** | Impact of Social Media on Aesthetic Dentistry: General Practitioners' Perspectives |
| **46** | The global reach of social media in oral and maxillofacial surgery |
| **47** | Online Professionalism of Facebook Usage in Dental Education: A Retrospective Exploration |
| **48** | Integrating digital scholarship in dental education: Why, what, and how? |
| **49** | Social media use, professional behaviors online, and perceptions toward e-professionalism among dental students |
| **50** | Implementation of Public Health Policies and Integration of Artificial Intelligence and Social Media in Dental Traumatology-Cornerstones for Effective Dental Trauma Management |
| **51** | Evaluating video-based lectures on YouTube for dental education |
| **52** | Orthodontics social media, perceptions of science- and non-science-based posts among orthodontists, dentists, students and laypeople |
| **53** | Online videos: The hidden curriculum |
| **54** | Social media and orthodontics: A commentary on a systematic review |
| **55** | Social media in health communication: A literature review of information quality |
| **56** | Social Media and Its Implications in Pediatric Dentistry |
| **57** | Role of Digital Media in Promoting Oral Health: A Systematic Review |
| **58** | Social Media Use by Dental Hygiene Educators |
| **59** | Social media and Dentistry: ethical and legal aspects |
| **60** | YouTube information about diabetes and oral healthcare |
| **61** | TikTok: An Opportunity for Antibiotic Education? |
| **62** | An analysis of dental articles in predatory journals and associated online engagement |
| **63** | The Modern and Digital Transformation of Oral Health Care: A Mini Review |
| **64** | Use of TikTok to improve the knowledge of Peruvian accreditation in Dentistry students |
| **65** | Promoting early childhood oral health and preventing early childhood caries on Instagram |
| **66** | Social media in dental education: a call for research and action |
| **67** | Characterizing the Content Related to Oral Health Education on TikTok |
| **68** | Dental Blogs, Podcasts, and Associated Social Media: Descriptive Mapping and Analysis |
| **69** | The quality of orthognathic surgery information on social media: A scoping review |
| **70** | Who is providing dental education content via YouTube? |
| **71** | American Twitter users revealed social determinant-related oral health disparities amid the COVID-19 pandemic |
| **72** | Social Media's Use and Impact on Oral Surgeons and Oral Surgery Residents |
| **73** | Social media patient testimonials in implant dentistry: information or misinformation? |
| **74** | Using Twitter for Teaching and Learning in an Oral and Maxillofacial Radiology Course |
| **75** | Dental trauma in social media-Analysis of Facebook content and public engagement |
| **76** | Is YouTube a useful tool for oral care in patients with Parkinson's disease? |
| **77** | Pattern of dental needs and advice on Twitter during the COVID-19 pandemic in Saudi Arabia |
| **78** | Revolution in modern teaching in dentistry since the appearance of the COVID-19 pandemic: A review |
| **79** | YouTube as an information source in paediatric dentistry education: Reliability and quality analysis |
| **80** | Association of dental caries with use of internet and social media among 12 and 15-year-olds |
| **81** | The Impact of Social Media on Professional Learning among Undergraduate Dental Students: A Cross-sectional Study |
| **82** | Youtube(TM) Content Analysis as a Means of Information in Oral Medicine: A Systematic Review of the Literature |
| **83** | YouTube, dentistry, and dental education |
| **84** | The Use of Instagram Among Dental Patients in Saudi Arabia |
| **85** | YouTube(™) as a source for patient education about the management of dental avulsion injuries |
| **86** | Exploring online oral health misinformation: a content analysis |
| **87** | Evaluation of Social Media Usage by Dental Practitioners of Pakistan for Professional Purposes - A Cross-Sectional Study |
| **88** | E-Professionalism among Dental Students from Malaysia and Finland |
| **89** | What do TikTok videos offer us about dental implants treatment? |
| **90** | The awareness and usage of orthodontic apps and social media by orthodontists in the UK: A questionnaire-based study |
| **91** | Yonder: New normal, dental risk, shared decision making in China, antidepressants on Instagram, and podcast of the month |
| **92** | COVID-19, social media use and anxiety: more complex than it might appear? |
| **93** | Dental Anxiety as a Risk Factor for Facebook Intrusion |
| **94** | The educational use of social networking sites among medical and health sciences students: a cross campus interventional study |
| **95** | Dentist-patient communication on dental anxiety using the social media: A randomized controlled trial |
| **96** | Determining the impact of orthodontic patients' characteristics on their usage and preferences of social media |
| **97** | #clearaligners and social media: An in-depth analysis of clear aligners' content on Instagram |
| **98** | Social media and orthodontics: A mixed-methods analysis of orthodontic-related posts on Twitter and Instagram |
| **99** | 10 ways dental practices can use social media |
| **100** | Social Media in the Dental School Environment, Part B: Curricular Considerations |
| **101** | Are dental x-rays safe? Content analysis of English and Chinese YouTube videos |
| **102** | Quo vadis, esthetic dentistry? Part II: Composite resin overtreatment and social media appeal |
| **103** | An Evaluation of the Usefulness of YouTube® Videos on Crown Preparation |
| **104** | Social media in adolescent health literacy education: a pilot study |
| **105** | The reach, influence and tenor of professional orthodontic societies on social media: A cross-sectional content analysis |
| **106** | Is YouTube(TM) an adequate source of oral hygiene education for orthodontic patients? |
| **107** | Periodontists' perceptions and attitudes toward the use of social media for professional purposes in Saudi Arabia |
| **108** | Orthodontic marketing through social media networks: the patient and practitioner's perspective |
| **109** | Social Media Use Behaviors and State Dental Licensing Boards |
| **110** | Is the information about orthodontics on Youtube and TikTok reliable for the oral health of the public? A cross sectional comparative study |
| **111** | Connectivity and Integration of Instagram(®) Use in the Lives of Dental Students and Professionals: A Country-Wide Cross-Sectional Study Using the InstaAA© Questionnaire |
| **112** | Is the quality of the orthodontic information in social media platforms reliable?: A systematic review |
| **113** | To what extent is oral and maxillofacial surgery educational content posted on Instagram? |
| **114** | Social Media Research Strategy to Understand Clinician and Public Perception of Health Care Messages |
| **115** | Social media as a learning tool in anatomy education from the perspective of medical and dental students |
| **116** | The Role of Social Media in Communication and Learning at the Time of COVID-19 Lockdown-An Online Survey |
| **117** | Use of Social Media to View and Post Dentistry-related Information in Bahrain: A Cross-Sectional Study |
| **118** | Influence of the use of social media on patients changing dental practice: a web-based questionnaire study |
| **119** | Public sense of dental implants on social media: A cross-sectional study based on text analysis of comments |
| **120** | Characterization of False or Misleading Fluoride Content on Instagram: Infodemiology Study |
| **121** | Looking good but tweeting bad? The social perception of orthodontic-related posts on Twitter and Instagram |
| **122** | YouTube™ as a source of information on oral habits |
| **123** | Social media use habits, and attitudes toward e-professionalism among medicine and dental medicine students: a quantitative cross-sectional study |
| **124** | Can a Social Media Application be a Valid Educational Tool for Oral and Maxillofacial Surgery Patients? |
| **125** | A survey of social media policies in U.S. dental schools |
| **126** | Social media and dentistry: some reflections on e-professionalism |
| **127** | Assessment of Indonesian-Language Orthodontics-Related YouTube Video as a Source of Information |
| **128** | Does Social Media Increase Perioperative Anxiety in Patients Undergoing Impacted Third Molar Surgery? |
| **129** | Using Instagram as a tool to enhance anatomy learning at two US dental schools |
| **130** | Dental fear and anxiety in children and adolescents: qualitative study using YouTube |
| **131** | Analysis of YouTube videos related to a child's first dental visit |
| **132** | YouTube™ as a source of information on extraction of third molars |
| **133** | Twitter communication of the UK public on dental health and care during a COVID lockdown: "My kingdom for a dentist" |
| **134** | Professional use of social media platforms by independent dental hygienists in the Netherlands: A quantitative study |
| **135** | Availability of 'Do-It-Yourself' orthodontics in the United Kingdom |
| **136** | Social networking website increases efficacy and engagement in a distance learning course about oral lesions |
| **137** | Reviewing the Role of Instagram in Education: Can a Photo Sharing Application Deliver Benefits to Medical and Dental Anatomy Education? |
| **138** | #Wisdomteeth: an analysis of 100 social media posts and a survey on patient perception |
| **139** | Factor analysis of risk perceptions of using digital and social media in dental education and profession |
| **140** | Emotional effect of the Covid-19 pandemic on oral surgery procedures: a social media analysis |
| **141** | The Effect Of Social Media On The Choice Of Dental Patients: A Cross-Sectional Study In The City Of Jeddah, Saudi Arabia |
| **142** | YouTube(™) for information on paediatric oral health instructions |
| **143** | Content Analysis of YouTube Videos on Radiographic Anatomy on Dental Panoramic Images |
| **144** | YouTube as a source of information on space maintainers for parents and patients |
| **145** | Are YouTube videos related to dental implant useful for patient education? |
| **146** | The relationship between Facebook behaviour and e-professionalism: A questionnaire-based cross-sectional study among Greek dental students |
| **147** | Social Media in the Dental School Environment, Part A: Benefits, Challenges, and Recommendations for Use |
| **148** | The risks and benefits of social media in dental foundation training |
| **149** | Social media impact on students' decision-making regarding aesthetic dental treatments based on cross-sectional survey data |
| **150** | A survey of US dental practices' use of social media |
| **151** | Social Media vs. Mass Media: Mitigating the Suspicion of Ulterior Motives in Public Health Communication |
| **152** | YouTube: a new way of supplementing traditional methods in dental education |
| **153** | Hashtag, like or tweet: a qualitative study on the use of social media among dentists in London |
| **154** | Content Analysis of YouTube Videos That Demonstrate Panoramic Radiography |
| **155** | Quality of information on Instagram about masseter botox injection for bruxism |
| **156** | The ethics of social media in dental practice: challenges |
| **157** | Does YouTube provide adequate information about orthodontic pain? |
| **158** | Oral health promotion and programming provided by Aboriginal Community Controlled Health Organisations in South Australia |
| **159** | Influence of social media platforms in dental education and clinical practice: A cross-sectional survey among dental trainees and professionals |
| **160** | Heutagogy through Facebook for the Millennial learners |
| **161** | YouTube™ as an information resource for orthognathic surgery |
| **162** | YouTube as a Source of Patient Information on Oral Manifestations of COVID-19: A Cross-Sectional Evaluation of Its Utility, Dependability, and Content |
| **163** | #Teeth&Tweets: the reach and reaction of an online social media oral health promotion campaign |
| **164** | YouTube™ as an information source for regenerative endodontic treatment procedures: Quality and content analysis |
| **165** | Using mobile multimedia platforms in teaching dental diagnosis |
| **166** | YOUTUBE videos on oral care of the organ or hematopoietic stem cell transplant patients |
| **167** | Analysis of Dentistry YouTube Videos Related To COVID-19 |
| **168** | Instant Messaging in Dental Education |
| **169** | Use of social media in dental schools: pluses, perils, and pitfalls from a legal perspective |
| **170** | YouTube use among dental students for learning clinical procedures: A multi-institutional study |
| **171** | Social media and professionalism: does the profession need to re-think the parameters of professionalism within social media? |
| **172** | Assessment of Reliability of YouTube Videos on Orthodontics |
| **173** | Reacting, Sharing, and Commenting: How Many Facebook Users Are Engaging with Posts Related to Dental Caries That Contain Misinformation? |
| **174** | Social media use, attitudes, behaviours and perceptions of online professionalism amongst dental students |
| **175** | Content quality and reliability of YouTube videos on oral appliance therapy for obstructive sleep apnea: A systematic analysis |
| **176** | Analysis of Fluoride-Free Content on Twitter: Topic Modeling Study |
| **177** | YouTube™ video content analysis on space maintainers |
| **178** | #implantology: A content analysis of the implant-related hashtags on Instagram |
| **179** | #CleftProud: A Content Analysis and Online Survey of 2 Cleft Lip and Palate Facebook Groups |
| **180** | Insights on the digitalisation of dental practices: A cross-sectional pilot study in Hesse |
| **181** | The 'five star' fallacy: an analysis of online reviews and testimonials of dental practices in Northern England |
| **182** | A cross-sectional study of exposure across social media platforms for the British Orthodontic Society retention awareness campaign: #HoldthatSmile |
| **183** | Exploring How People Interact With Dental Misinformation on Social Media: A Cross-Sectional Study |
| **184** | The impact of social media on dental practice promotion and professionalism amongst general dental practitioners and specialists in KSA |
| **185** | Differences between doctors of medicine and dental medicine in the perception of professionalism on social networking sites: the development of the e-professionalism assessment compatibility index (ePACI) |
| **186** | YouTube as a source of information on adult orthodontics: a video analysis study |
| **187** | Quality of YouTube TM videos on dental implants |
| **188** | Reliability of information on YouTube™ regarding pediatric dental trauma |
| **189** | Defining Potentially Unprofessional Behavior on Social Media for Health Care Professionals: Mixed Methods Study |
| **190** | YouTube™ as a Source of Information for Patients Regarding Dental Implant Failure: A Content Analysis |
| **191** | Exploring predoctoral dental student use of YouTube as a learning tool for clinical endodontic procedures |
| **192** | Evaluation of the reliability and accuracy of YouTube™ and TikTok™ contents about storage media for avulsed teeth: A cross-sectional study |
| **193** | Factors That Affect Saudi Population Preferences Toward Their Dentist |
| **194** | Analysis of Quality, Usefulness, Reliability, Visibility, and Popularity of Videos about Dental Caries on YouTube: A Cross-sectional Analysis |
| **195** | The effects of images posted to social media by orthodontists on public perception of professional credibility and willingness to become a client |
| **196** | Discourse about human papillomavirus (HPV)-associated oropharyngeal cancer (OPC) on Twitter: Lessons for public health education about OPC and dental care |
| **197** | YouTube™ as a source of tooth avulsion information: A video analysis study |
| **198** | Examination of social networking professionalism among dental and dental hygiene students |
| **199** | Is social media the way to empower patients to share their experiences of dental care? |
| **200** | How compliant are dental practice Facebook pages with Australian health care advertising regulations? A Netnographic review |
| **201** | Perceptions of professional social media interaction with patients and faculty members - a comparative survey among dental students from Malaysia and Finland |
| **202** | Self-perception of personal oral health in Saudi population: a social media approach |
| **203** | What are dental professionals posting on Facebook? A cross-sectional content analysis |
| **204** | Quantitative and qualitative analyses of orthodontic-related videos on YouTube |
| **205** | YouTube as a source of information about orthodontic clear aligners |
| **206** | Quality of Information on YouTube™ about Rapid Maxillary Expansion |
| **207** | The use and ethics of dental photography and social media at an oral healthcare training centre in South Africa |
| **208** | Promoting Oral Health Using Social Media Platforms: Seeking Arabic Online Oral Health Related Information (OHRI) |
| **209** | Nature and Diffusion of COVID-19-related Oral Health Information on Chinese Social Media: Analysis of Tweets on Weibo |
| **210** | The use of social media for professional purposes among dentists in Saudi Arabia |
| **211** | Advertising and facial aesthetics in primary care: how compliant are practice websites and social media with published guidance? |
| **212** | Facebook as a learning environment for teaching medical emergencies in dental practice |
| **213** | Dento-legal Aspects of Advertising and the Use of Social Media by Dental Professionals |
| **214** | Does YouTube™ offer high quality information? Evaluation of accelerated orthodontics videos |
| **215** | Dental Care in the Arab Countries During the COVID-19 Pandemic: An Infodemiological Study |
| **216** | Community water fluoridation online: an analysis of the digital media ecosystem |
| **217** | Connecting With Your Dentist on Facebook: Patients' and Dentists' Attitudes Towards Social Media Usage in Dentistry |
| **218** | A Social Media Content Analysis of Dental Health Information Involving the Use of Miswak (Salvadora persica) Chewing Stick on YouTube™ |
| **219** | Determining the usage of social media for medical information by the medical and dental students in northern Jordan |
| **220** | YouTube Videos: A Learning Tool for Periodontology Education |
| **221** | Topical Fluoride Applications Related Posts Analysis on Twitter Using Natural Language Processing |
| **222** | The Influence of Social Network Content on the Perception of Smiles-A Randomized Controlled Trial |
| **223** | Orthodontic retention and retainers: Quality of information provided by dental professionals on YouTube |
| **224** | Users' passivity in accessing digested scientific evidence through social media: cross-sectional insights |
| **225** | An analysis of YouTube videos as educational resources for dental practitioners to prevent the spread of COVID-19 |
| **226** | The adoption of social media and social media marketing by dentists in South Africa |
| **227** | Quality of Patient-Centered eHealth Information on Erosive Tooth Wear: Systematic Search and Evaluation of Websites and YouTube Videos |
| **228** | Perceptions of e-professionalism among dental students: a UK dental school study |
| **229** | Evaluating YouTube as a Patient Information Source for the Risks of Root Canal Treatment |
| **230** | Facebook advertising: 5 Tools dental practices must use to generate results |
| **231** | Evaluation of the quality of YouTube™ videos about pit and fissure sealant applications |
| **232** | Content analysis of fluoride-related posts on Instagram |
| **233** | Current Status of Instagram Utilization by Oral and Maxillofacial Surgery Residency Programs: A Comparison With Related Dental and Surgical Specialties |
| **234** | Use of social media by dental educators |
| **235** | An Innovative Use of Twitter to Disseminate and Promote Medical Student Scholarship During the COVID-19 Pandemic: Usability Study |
| **236** | Analysis of highly tweeted dental journals and articles: a science mapping approach |
| **237** | YouTube as a learning modality for clinical procedures among medical and dental students: A study in public sector teaching institutes |
| **238** | The use of social media in dental hygiene programs: a survey of program directors |
| **239** | Analysis of tweets on toothache during the COVID-19 pandemic using the CrystalFeel algorithm: a cross-sectional study |
| **240** | YouTube as an information source for instrument separation in root canal treatment |
| **241** | Pediatric Dental Residency Program Directors' Perspectives on and Use of Social Media for Resident Selection and Education |
| **242** | Assessing the quality of YouTube™ videos on fixed dental implant home-care and maintenance protocols |
| **243** | YouTube videos on gingival grafting procedures: Content and quality analysis |
| **244** | Social media and professionalism: a retrospective content analysis of Fitness to Practise cases heard by the GDC concerning social media complaints |
| **245** | Accounts of bullying on Twitter in relation to dentofacial features and orthodontic treatment |
| **246** | Lingual orthodontic treatment: A YouTube™ video analysis |
| **247** | Unraveling dental caries misinformation: Identifying predictive factors for engagement on Instagram |
| **248** | Differences between Doctors of Dental Medicine and Doctors of Medicine Awareness of Their Online Image and Perception Concerns: a Quantitative Cross-Sectional Study |
| **249** | Quality of information on YouTube about artificial intelligence in dental radiology |
| **250** | Orthodontic Elastics: A Multivariable Analysis of YouTube(TM) Videos |
| **251** | YouTube as a source for parents' education on early childhood caries |
| **252** | A school-wide assessment of social media usage by students in a US dental school |
| **253** | YouTube as a source of information about rubber dam: quality and content analysis |
| **254** | Innovative Integration of Facebook Groups in Biomaterials Course: Perception of Dental Students |
| **255** | 'Have you seen what is on Facebook?' The use of social networking software by healthcare professions students |
| **256** | Orthodontic YouTube™ videos made by patients for patients: What are they about and are they accurate? |
| **257** | Decoding early childhood caries: an in-depth analysis of YouTube videos for effective parental education |
| **258** | A mixed-method exploration of #vapingcessation videos on TikTok |
| **259** | Reach of Messages in a Dental Twitter Network: Cohort Study Examining User Popularity, Communication Pattern, and Network Structure |
| **260** | Evaluation of the usefulness and quality of YouTube™ videos about children's electric toothbrushes |
| **261** | Assessment of the quality of oral biopsy procedure videos shared on YouTube |
| **262** | Measuring the social impact of dental research: An insight into the most influential articles on the Web |
| **263** | Exploring Public Perceptions of Dental Care Affordability in the United States: Mixed Method Analysis via Twitter |
| **264** | A Critical Review of YouTube Videos on the Socket-shield Technique: A Content-quality Analysis |
| **265** | Differences in Emotional and Pain-Related Language in Tweets About Dentists and Medical Doctors: Text Analysis of Twitter Content |
| **266** | Analysis of YouTube videos as a source of information about dentin hypersensitivity |
| **267** | Are YouTube videos about skull bone anatomy useful for students? |
| **268** | An assessment of professionalism on students' Facebook profiles |
| **269** | Assessment of dentists' behaviour on the use of patients' images |
| **270** | Medical Institutions and Twitter: A Novel Tool for Public Communication in Japan |
| **271** | A short-term evaluation of oral hygiene education methods in fixed orthodontics patients: a randomized clinical trial comparing assistant training, software, and social media |
| **272** | Use of YouTube™ as a self-directed learning resource in oral surgery among undergraduate dental students: a cross-sectional descriptive study |
| **273** | Evaluation of the Quality of Peri-implantitis Videos on YouTube |
| **274** | Influence of Social Media towards the Selection of Hollywood Smile among the University Students in Riyadh City |
| **275** | The availability of open-access videos offered by dental schools |
| **276** | The unbearable emptiness of tweeting-About journal articles |
| **277** | YouTube as a source of information about pulpotomy and pulp capping: a cross sectional reliability analysis |
| **278** | Assessment of the reliability of YouTube™ videos about zirconia crowns in pediatric dentistry |
| **279** | Uncovering a pseudoscience: an analysis of 'biological dentistry' Instagram posts |
| **280** | "How I whiten my teeth": YouTube™ as a patient information resource for teeth whitening |
| **281** | Identifying Risk Factors Affecting the Usage of Digital and Social Media: A Preliminary Qualitative Study in the Dental Profession and Dental Education |
| **282** | The Use of Social Media by Dental Students for Communication and Learning: Two Viewpoints: Viewpoint 1: Social Media Use Can Benefit Dental Students' Communication and Learning and Viewpoint 2: Potential Problems with Social Media Outweigh Their Benefits for Dental Education |
| **283** | Public health surveillance of dental pain via Twitter |
| **284** | YouTube video analysis as a source of information for patients on impacted canine |
| **285** | The use of digital technologies in dental practices in Switzerland: a cross-sectional survey |
| **286** | YouTube as a patient-information source for root canal treatment |
| **287** | An appraisal of the current and potential value of web 2.0 contributions to continuing education in oral implantology |
| **288** | Information-seeking behaviors and barriers to the incorporation of scientific evidence into clinical practice: A survey with Brazilian dentists |
| **289** | Invisalign treatment from the patient perspective: A Twitter content analyses |
| **290** | YouTube as an information source for bleeding gums: A quantitative and qualitative analysis |
| **291** | Does time matter? WhatsApp vs electronic mail for dental education. A pilot study |
| **292** | A qualitative analysis of orthodontic-related posts on Twitter |
| **293** | The online attention to orthodontic research: an Altmetric analysis of the orthodontic journals indexed in the journal citation reports from 2014 to 2018 |
| **294** | The Impact of the COVID-19 Epidemic on Orthodontic Patients in China: An Analysis of Posts on Weibo |
| **295** | Influence of WhatsApp and electronic mail reminders on oral hygiene compliance of orthodontic patients using planimetry : A randomized clinical trial |
| **296** | The ethics of social media in dental practice: ethical tools and professional responses |
| **297** | Use of information and communication technology amongst New Zealand dental students |
| **298** | Children's Toothbrushing Practices Recommended on the Internet by Pediatric Dentistry Associations |
| **299** | Reliability of Educational Content Videos in YouTube(TM) about Stainless Steel Crowns |
| **300** | Informational value and bias of videos related to orthodontics screened on a video-sharing Web site |
| **301** | Exploring the impact of digital professionalism awareness training on dental undergraduate students |
| **302** | Analysis of Cyberincivility in Posts by Health Professions Students: Descriptive Twitter Data Mining Study |
| **303** | What dental educators need to understand about emerging technologies to incorporate them effectively into the educational process |
| **304** | Student's Perception of the Impact of E-learning on Dental Education |
| **305** | Does YouTube(TM) Offer High-Quality Information About Nasoalveolar Molding? |
| **306** | Use of Social Media for Out-of-Class Communication to Enhance Learning: A pilot study |
| **307** | Dental students' learning attitudes and perceptions of YouTube as a lecture video hosting platform in a flipped classroom in Korea |
| **308** | Medical reliability of a video-sharing website: The gingival recession model |
| **309** | Reliability of information in YouTube™ videos on types of root resorption and related stimulating factors |
| **310** | Are YouTube videos on complete arch fixed implant-supported prostheses useful for patient education? |
| **311** | Knowledge management systems for oral health in developing and developed countries |
| **312** | Health impacts of Facebook usage and mobile texting among undergraduate dental students: it's time to understand the difference between usage and an excessive use |
| **313** | Quality of YouTube videos on botulinum toxin management in bruxism, assessed using the DISCERN instrument |
| **314** | "My Invisalign experience": content, metrics and comment sentiment analysis of the most popular patient testimonials on YouTube |
| **315** | Orthodontic practice marketing: The orthodontist and laypeople's perspective |
| **316** | Analysis of the Informational Content of Turkish Videos on YouTube About Tooth Brushing |
| **317** | Critical evaluation of YouTube videos regarding the all-on-4 dental implant treatment concept: A content-quality analysis |
| **318** | Twitter analysis of the orthodontic patient experience with braces vs Invisalign |
| **319** | Effect of social media in improving knowledge among patients having fixed appliance orthodontic treatment: A single-center randomized controlled trial |
| **320** | Patient-Centered Communication: Exploring the Dentist's Role in the Era of e-Patients and Health 2.0 |
| **321** | Experiences Of Individuals Concerning Combined Orthodontic and Orthognathic Surgical Treatment: A Qualitative Twitter Analysis |
| **322** | Should 'fitness to practise' include safeguarding the reputation of the profession? |
| **323** | Making impressions count: An evaluation of the quality of information provided by orthodontic practices in London in response to the COVID-19 pandemic |
| **324** | How accessible are you? A hospital-wide audit of the accessibility and professionalism of Facebook profiles |
| **325** | Tweeting about pain: comparing self-reported toothache experiences with those of backaches, earaches and headaches |
| **326** | Social media use by orthodontic patients |
| **327** | Social media: the word of mouth revolution |
| **328** | Occlusal device therapy for sleep bruxism: Analysis of educational value, clarity, reliability, understandability, and actionability of information of content on YouTube |
| **329** | Comprehensive Analysis of Factors Influencing Patients' Preferences and Attitudes toward Dental Treatment Choices |
| **330** | Social media policy in other orqanizations |
| **331** | Assessment of usefulness and reliability of YouTube™ videos on denture care |
| **332** | Evaluation of the Quality of Educational Content of YouTube Videos on Silver Diamine Fluoride |
| **333** | Content quality and reliablity of YouTube™ videos as a source of information about good oral hygiene practices in adults |
| **334** | Development and validation of scale for measuring attitudes towards e-professionalism among medical and dental students: SMePROF-S scale |
| **335** | YouTube™ Videos as a Source of Information on Necrotizing Gingivitis: A Content-Quality Analysis |
| **336** | Evolving perspectives in dental marketing: A study of Jordanian dentists' attitudes towards advertising and practice promotion |
| **337** | Plugged in: social media in the dental profession |
| **338** | Orthodontic treatment with miniscrew anchorage: Analysis of quality of information on YouTube |
| **339** | Conflicting demands that dentists and dental care professionals experience when using social media: a scoping review |
| **340** | Evaluation of YouTube videos for patients' education on periradicular surgery |
| **341** | Anxiety among dental professionals and its association with their dependency on social media for health information: insights from the COVID-19 pandemic |
| **342** | Influence of social media on the esthetic perception of the lip profile of orthodontic patients |
| **343** | Learning Clinical Procedures Through Internet Digital Objects: Experience of Undergraduate Students Across Clinical Faculties |
| **344** | Adverse health effects and unhealthy behaviors among dental undergraduates surfing social networking sites |
| **345** | Evaluation of the Quality and Reliability of YouTube(TM) Videos Created by Orthodontists as an Information Source for Clear Aligners |
| **346** | Sensationalist social media usage by doctors and dentists during Covid-19 |
| **347** | The impact of social media on the selection of dentists based on their social media presence among residents of Vojvodina, Serbia: a cross-sectional study |
| **348** | Critical appraisal of YouTube videos regarding peri-implant diseases and conditions: A cross-sectional analysis |
| **349** | Does YouTube™ Provide Adequate Information on Oral Health During Pregnancy? |
| **350** | Clarity of publications on HPV in Instagram profiles of official health agencies in Brazil |
| **351** | The role of social media for patients with temporomandibular disorders: A content analysis of Reddit |
| **352** | Keyword Trends for Mother-Child Oral Health in Korea Based on Social Media Big Data from Naver |
| **353** | Impact of Coronavirus disease 2019 on patients with toothache: Analysis of tweets on Weibo |
| **354** | Social Media Impact on Self-Perceived Oral Health Practices Among Patients Visiting Tertiary Care Hospital in Lucknow: A Cross-Sectional Study |
| **355** | Measuring e-Professional Behavior of Doctors of Medicine and Dental Medicine on Social Networking Sites: Indexes Construction With Formative Indicators |
| **356** | Assessment of the educational value of endodontic access cavity preparation YouTube video as a learning resource for students |
| **357** | The Application of a Case-Based Social Media-Assisted Teaching Method in Cariology Education：Comparative Study |
| **358** | Exploring the advantages of using social network sites (SNSs) in dental medicine organisations |
| **359** | The impact of school-based social media and online technology on oral health education for individuals with disability |
| **360** | A Scoping Review of eProfessionalism in Healthcare Education Literature |
| **361** | Looking Beyond Traditional Metrics in Orthodontics: An Altmetric Study on the Most Discussed Articles on the Web |
| **362** | Orthodontics in an online community: A computational analysis of r/Braces subreddit |
| **363** | User requirement gathering for online oral health education module development: Exploring parental perspective |
| **364** | Self-reported dental treatment needs during the COVID-19 outbreak in Brazil: an infodemiological study |
| **365** | Psychophysiological Reactions of Internet Users Exposed to Fluoride Information and Disinformation: Protocol for a Randomized Controlled Trial |
| **366** | The use of internet platforms for oral health information and associated factors among adolescents from Jakarta: a cross sectional study |
| **367** | The use of information and communication technologies in Latin American dentists: a cross-sectional study from Ecuador |
| **368** | Effectiveness of a message service on child oral health practice via a social media application: A randomized controlled trial |
| **369** | A review of the effects of oral health media hype on clients' perception of treatment |
| **370** | Development and validation of self-assessment instrument to measure the digital professionalism of healthcare professionals using social media |
| **371** | Online audiovisual resources for learning the disinfection protocol for dental impressions: A critical analysis |
| **372** | Improvement of oral health knowledge and behavior of diabetic patients: an interventional study using the social media |
| **373** | Information seeking behaviour of dental trauma patients and their parents |
| **374** | Viewing of clinical cases on social media by dentists: A cause of motivation or dissatisfaction? |
| **375** | Comparative assessment of attitudes among medical and dental professionals in Saudi Arabia toward e-professionalism using the SMEPROF-S scale |
| **376** | Infodemiology for oral health and disease: A scoping review |
| **377** | Are dentists interested in the oral-systemic disease connection? A qualitative study of an online community of 450 practitioners |
| **378** | Effectiveness of social media based oral health promotion programme among 18-20 year old city college students - A comparative study |
| **379** | Legal issues in digital oral health: a scoping review |
| **380** | Arabic Web-Based Information on Oral Lichen Planus: Content Analysis |
| **381** | The Association of Social Media Videos and Patients' Preoperative Anxiety |
| **382** | Impact of social media on the oral hygiene habits of children and adolescents: a randomized controlled clinical trial |
| **383** | The relation between social media mentions and academic citations in orthodontic journals: A preliminary study |
| **384** | Using Twitter to promote #orthodontic research and clinical innovations: Practice and challenges |
| **385** | How far do oral lectures at European Orthodontic Society congresses reach? A comparison of abstract publication rates, article citations, and social media mentions |
| **386** | The evolving role of social media in paediatric dentistry: A narrative review |
| **387** | Can Social Media be Professional Too? |
| **388** | Sharing endodontic research on social media platforms: Is it effective? A 10-year timeline analysis |
| **389** | Knowledge, Attitude, and Practices of Dentists in Maharashtra Regarding Sharing Patients’ Data on Social Media: A Questionnaire-Based Study |
| **390** | Is Trending Social Media Platforms a Newfangled Language of Oral Health Promotion? A Short Communication |
| **391** | Influence of Search Engines and Social Media on Dental Patients’ Health Information Seeking: A Cross-sectional Study |
| **392** | Use of social media by dental students: A comparative study |
| **393** | The Social Impact of Dental Implant Research: an Altmetric Analysis |
| **394** | The Role of Social Media on Dental Education and Oral Health: A Focus on Instagram |
| **395** | Prevalence of Social Media Use for Dental Information Among Dental Professionals |
| **396** | INFLUENCE OF SOCIAL MEDIA TOWARDS AESTHETIC DENTISTRY: PERSPECTIVE OF PATIENTS IN UKM KL DENTAL CLINIC |
| **397** | Perceptions towards orthodontic marketing through social media among young adults seeking orthodontic treatment: a qualitative study |
| **398** | What are orthodontic residents perusing on social media? A cross-sectional survey |
| **399** | Effects of Social Media on Consumer Information Seeking and Purchase Intention of Oral Health Care Products Among Adults |
| **400** | Social Media and Dental Practitioner's Knowledge of Misinformation, Infodemic and Fact-Checking on Dental Information |
| **401** | Does Social Media Affect a Patient’s Decision to Undergo Orthognathic Surgery? |
| **402** | Are Social Media Platforms Appropriate Sources of Information for Patients Regarding the Topic of Facial Trauma? |
| **403** | Preferences, perception and impact of using dental social media in Kuwait |
| **404** | Influence of social media and corrected smile photographs in patients with malocclusion |
| **405** | Direct and indirect restorations in posterior sectors: literature vs social media; [Restauri diretti e indiretti nei settori posteriori: confronto letteratura-social media] |
| **406** | Family doctors’ and dentists’ access and dissemination of medical information through social media |
| **407** | The Trend of Children's Dental Health Information-Seeking Behaviors on Social Media: A narrative review |
| **408** | The Impact of Social Media Posts Concerning Esthetic Dentistry on Self-Esteem and Satisfaction Among Adults in Saudi Arabia |
| **409** | DENTAL MISCONCEPTIONS IN SOCIAL MEDIA ACCOUNTS: YOUTUBE AND INSTAGRAM APPLICATIONS AMONG FLUORIDE TOXICITY, BLEACHING AND, WATERJET |
| **410** | The use of social media in the patient-doctor relationship: The case of Iraq |
| **411** | Trialling a novel socially-distanced teaching approach for OMFS dental core trainees: case-based discussions through WhatsApp© |
| **412** | Oral and Maxillofacial Surgery Journals’ Presence on Social Media: An Adaptation to Enhance Publication Readership and Interdisciplinary Collaboration? |
| **413** | Effect of Education Based on Trans-Theoretical Model in Social Media on Students with Gingivitis; a Randomized Controlled Trial |
| **414** | The effect of chairside verbal instructions matched with instagram social media on oral hygiene of young orthodontic patients: A randomized clinical trial |
| **415** | Parental and provider perspectives on social media about ankyloglossia |
| **416** | An Update on Instagram Utilization by Oral and Maxillofacial Surgery Residency Programs: The Social Media Big Bang |
| **417** | Use of Social Media in Dental Education: A Single Institutional Study |
| **418** | Social media as a tool in dental public health |
| **419** | Social media and orthodontics: An analysis of orthodontic-related posts on instagram |
| **420** | Impact of social media on dental students in Chennai |
| **421** | Insights on using social media in dental education: A cross-sectional study in Saudi Arabia |
| **422** | Effective Use of Social Media |
| **423** | Social media utilization among dental practitioner in Riyadh, Saudi Arabia |
| **424** | The dangers of social media and young dental patients' body image |
| **425** | What are the potential ethical considerations of a dentist connecting, that is, “friending,” on social media with a patient of record? |
| **426** | Social networking in dentistry: A review |
| **427** | Adherence to professionalism and ethical practice on social media among dentists in nairobi |
| **428** | Attitudes toward social media among practicing dentists and dental students in clinical years in Saudi Arabia |
| **429** | Analysis of Doctor-Patient Relationship in Social Media: the Case of Greece |
| **430** | Instagram as a social media tool about orthognathic surgery |
| **431** | Effects of social media on cognitive behavior among dental graduates |
| **432** | #JawSurgery: Analysis of social media use in orthognathic surgery patients |
| **433** | Older and wiser? First year BDS graduate entry students and their views on using social media and professional practice |
| **434** | Social networks in dental training: Opinion of students from a Brazilian university; [Redes sociais na aprendizagem em odontologia: Opinião dos estudantes de uma universidade brasileira]; [Redes sociales en el aprendizaje de la odontología: Opinión de los estudiantes de una universidad brasileña] |
| **435** | Dental burnout - Is social media a help or hindrance? |
| **436** | Using online social networks for increasing health literacy on oral health |
| **437** | How social media meet patients’ questions: YouTubE™ review for children oral thrush |
| **438** | Social Media - What dentists need to be aware of |
| **439** | Harness social media to grow your practice |
| **440** | Focus On: Social Media |
| **441** | Usage of Social Media by Medical and Dental Students at Nishtar Medical College, Multan, Pakistan |
| **442** | The dos and don'ts of social networking in dentistry |
| **443** | Being professional in the social media world. |
| **444** | Social media: why your practice needs to pay attention to digital moms. |
| **445** | The business of social media |
| **446** | Community water fluoridation on the Internet and social media. |
| **447** | Educational Implications of a Social Networking Application, Twitter™, for Anatomical Sciences |
| **448** | Kinship--king's social harmonisation project. Pilot phase of a social network for use in higher education (HE) |
| **449** | Top five reasons dentists will not use social media. |
| **450** | Modern dental practice for the dentists in the UAE using social networking tools |
| **451** | Social media marketing still subject to advertising rules. |
| **452** | Social media basics for orthodontists |
| **453** | Use of social networking for dental hygiene program recruitment. |
| **454** | Grow your practice using social media: if you aren't online, do patients know you exist? |
| **455** | Social media in the health care provider office. |
| **456** | Management & marketing: orthodontic marketing through online social networks. |
| **457** | Five social media survival tips for dentists. |
| **458** | Clarify your online marketing objectives before seeking social media status. |
| **459** | Tweets on water fluoridation |
| **460** | Analyzing the relationship between Altmetric score and literature citations in the Implantology literature |
| **461** | Exploring interactions between dental hygiene faculty and current undergraduate dental hygiene students on facebook |
| **462** | YouTubeTM as a supplemental learning source for undergraduate dental students |
| **463** | Comparison of YouTube and YouTube Shorts videos about clear aligners |
| **464** | YouTube™ Videos about Silver Diamine Fluoride in Pediatric Dentistry as a Source of Information: A Cross Sectional Analysis |
| **465** | Bruxism treatment on Youtube: evaluating reliability and information accuracy |
| **466** | Is it safe to learn about vital pulp capping from YouTube™ videos? A content and quality analysis |
| **467** | Does YouTube Provide High-Quality Information for Patients Regarding Night Guards, Especially for Over-the-Counter Ones? |
| **468** | Assessment of the Quality of Sinus Elevation with Lateral Window Approach Procedure Videos on YouTube: A Content-quality Analysis |
| **469** | Does the content quality of YouTube videos about aligners differ from the perspectives of dentists and orthodontists? |
| **470** | Quality of free gingival graft content in youtube videos: Usability in patient information and student education |
| **471** | Evaluation of YouTube as an information source for denture care |
| **472** | YouTube™ as a Tool to Teach Dentists about Molar Incisor Hypomineralization (MIH): Analysis of Quality Content |
| **473** | Assessment of the content and quality of YouTube videos related zygomatic implants: A content–quality analysis |
| **474** | YouTube™ as a source of information about dental sleep medicine |
| **475** | How reliable are YouTube videos on laser-assisted surgical treatment of the gummy smile? |
| **476** | Public Response to an Oral Health Promotion Disseminated Via YouTube during the COVID-19 Pandemic: A Study in an Indonesian Population |
| **477** | Quality of Tooth-Whitening Videos Available on YouTube |
| **478** | Assessment of reliability and information quality of YouTube videos about root canal treatment after 2016 |
| **479** | Analyses of Youtube Videos on Botox Treatment of Gummy Smile |
| **480** | YouTube™ for information on paediatric oral health instructions |
| **481** | Use of YouTube as a Learning Modality for Clinical Procedures among Dental Students in Riyadh, Saudi Arabia—A Cross-Sectional Study |
| **482** | Youtube™ Content Analysis as a Means of Information in Oral Medicine: A Systematic Review of the Literature |
| **483** | Is YouTubeTM a reliable source of information regarding ultra-thin ceramic laminates? |
| **484** | Is YouTube® Adequate as a Source of Patient Information for Intravenous Sedation in Dentistry? |
| **485** | Credibility of YouTube™ videos on root canal treatment in children |
| **486** | Are YouTube videos on regenerative endodontic procedure reliable source for patient edification? |
| **487** | Is YouTube an adequate patient resource about orthodontic retention? A cross-sectional analysis of content and quality |
| **488** | Youtube tm videos as a source of information on clear retainers |
| **489** | YouTube™ quality as a source for parent education about the oral hygiene of children |
| **490** | YouTube™ as a source for patient education about the management of dental avulsion injuries |
| **491** | Would YouTube a reliable source of information about dental bleaching in Brazil?; [Seria o YouTube uma fonte confiável de informação sobre clareamento dental no Brasil?] |
| **492** | Fluoride-related YouTube videos: A cross-sectional study of video contents by upload sources |
| **493** | Potential patient education of YouTube videos related to wisdom tooth surgical removal |
| **494** | Instagram as a Pedagogical Tool in Pediatric Dentistry and Ortodontic Courses |
| **495** | Examination of Information Quality on Public Instagram Profiles Regarding Botulinum Toxin for Bruxism: A Study in São Paulo State, Brazil |
| **496** | QUALITY AND INTERACTION LEVELS OF INSTAGRAM POSTS RELATED TO ORTHODONTIC #CLEARALIGNERS |
| **497** | Analyzing Content and Information Quality of Instagram® Posts About #teethwhitening; [#Diş Beyazlatma Hakkında Instagram® Gönderilerinin İçerik ve Bilgi Kalitesinin Analizi] |
| **498** | Main uses of Instagram in oral health research–A scoping review |
| **499** | An Analysis of Instagram Posts about Genioplasty |
| **500** | Email vs. Instagram recruitment strategies for online survey research |
| **501** | #Dentalpain: What do the brazilian Instagram® users want to mean? |
| **502** | The Perception and Usage of Fake Braces: Twitter Content Analysis |
| **503** | From textbooks to TikTok—The digital future of the dental world |
| **504** | Can TikTok Provide Reliable Information about Orthodontics for Patients? |
| **505** | Comparison of WhatsApp® and Face-to-Face case based learning in undergraduate dental students: A randomized controlled trial |
| **506** | WhatsApp as a Tool in Blended Learning in Dental Education |
| **507** | The effectiveness of education using whatsapp video calls on dental health knowledge among children aged 8-10 years |
| **508** | Whatsapp as a digital tool in educational intervention on oral health in Peruvian children during COVID-19 |
| **509** | A Comparative Analysis of the Impact of Audiovisual and Leaflets through Whatsapp as Oral Health Promotion Media on Adolescents' Knowledge of Oral Health |
| **510** | Students’ perception of WhatsApp usage as an additional learning aid at a Dental College in Pakistan |
| **511** | Inducting undergraduate medical students via WhatsApp-based multiple mini interviews during COVID-19 pandemic |
| **512** | Whatsapp as an E-learning tool of dental radiograph interpretation among dental undergraduates - A pilot study |
| **513** | Implementing post-orthodontic compliance among adolescents wearing removable retainers through whatsapp: A pilot study |
| **514** | French dentists' communication quality on clinical oral dermatology cases in Facebook groups: a cross-sectional study |
| **515** | Perceptions on Oral Ulcers From Facebook Page Categories: Observational Study |
| **516** | Communicating with patients through Facebook: The case of dental healthcare services |
| **517** | Facebook and dental education: What do students like? |
| **518** | Orthognathic surgery: Outcome in a facebook group |
| **519** | Practical tips for managing LinkedIn and Facebook (on top of everything else). |
| **520** | Is Online Video a Suitable Source to Obtain Sufficient and Useful Information About Peri-Implantitis? |
| **521** | Pre-recorded versus online Zoom lectures during COVID-19 pandemic: a cross-sectional study from a Private Medical and Dental College Lahore, Pakistan |
| **522** | Digital Entrepreneurship via Sustainable Online Communication of Dentistry Profession, Oradea, Romania: A Longitudinal Analysis |
| **523** | Semantic Analysis of Online Dentist Review: Toward Assessing Safety and Quality of Dental Care |
| **524** | The online attention to oral cancer research: An Altmetric analysis |
| **525** | Parents’ online discussions about children’s dental caries: A critical content analysis |
| **526** | Comparative analysis of user-generated online yelp reviews for periodontal practices in multiple metropolitan markets |
| **527** | Digital marketing in dentistry and ethical implications |
| **528** | Perceived Barriers in Digitalizing Oral Health Promotion: Phenomenological Study among Malaysian Dental Public Health Specialists |
| **529** | DESIGN OF MARKETING PLAN FOR DENTAL HOSPITALS IN THE DIGITAL ERA |
| **530** | Influence of digital media in the oral health education of mother-child pairs: study protocol of a parallel double-blind randomized clinical trial |
| **531** | Awareness and preferred mode of getting information on first aid management of avulsed permanent teeth: Survey of Nigerian mothers |
